# Analysis of Further Education Students’ Attitudes Regarding Environmental Pollution. A Case Study in Granada

**DOI:** 10.3390/ijerph16060905

**Published:** 2019-03-13

**Authors:** Francisco Javier Hinojo Lucena, Inmaculada Aznar Díaz, María Pilar Cáceres Reche, Juan Manuel Trujillo Torres, Gerardo Gómez García

**Affiliations:** Department of Didactics and School Organization, University of Granada, 18071 Granada, Spain; fhinojo@ugr.es (F.J.H.L.); iaznar@ugr.es (I.A.D.); caceres@ugr.es (M.P.C.R.); jttorres@ugr.es (J.M.T.T.)

**Keywords:** attitudes, pollution, Further Education, environmental education, sustainable development

## Abstract

Pollution is shown as the environmental challenge, which has the greatest impact on global climate change. Faced with this situation, numerous environmental summits agree on the fact that Environmental Education needs to be implemented within the different disciplines and educational institutions. Therefore, Further Education must foster the research and management of environmental education with the aim of developing responsible citizens with sustainable attitudes. Based on this idea, this paper aimed to analyse the attitudes in Further Education students towards different situations and habits linked to pollution, as well as some of its varied typologies (chemical pollution, acoustic pollution and management of solid urban waste and rubbish). To achieve this, a sample of 307 students from different degrees of Preschool and Primary Education was included, using a questionnaire as a measuring instrument. The methodology of the study was both descriptive, through the analysis of its measures, and inferential, with the preparation of a confirmatory conceptual model through the structural equation model (SEM). Results revealed that students are highly concerned about the different situations proposed, and that the predictive model forges strong correlations between the four variables of the study. Hence, the study focused on the idea of trying to enhance environmental awareness in the groups of students from different educational phases, to subsequently foster the implementation of specific actions aimed at preserving and conserving natural resources, and to guide society towards sustainable development.

## 1. Introduction

The environmental crisis and raising awareness of it have been areas of concern, not only for experts in Ecology and caring for the biosphere, but also for the educational community. Exponentially increasing phenomena, such as pollution in its different derivations, are a real and growing issue whose consequences are included in climate change as a global concern. Such a state of the planet is the result of numerous anthropic actions that are having an impact on the environment, of which the population is not aware. Consequently, these actions are continuously damaging the biosphere [1].

All these ideas have been challenged in different international summits on the environment over the years (Stockholm, 1972; Rio de Janeiro, 1992; New York, 2002; Johannesburg, 2002; Copenhagen, 2009), which have resulted in certain action protocols, as is the case with Kyoto (approved in 1997 and came into force in 2005), linked to reducing greenhouse gas emissions (CO_2_), or the Stockholm Convention (signed in 2001 and effective from 2004), associated with restricting the pollution caused by persistent organic pollutants (POPs). Among the main threads in these reports, the importance of Environmental Education (hereinafter EE) is seen as the key focus of human awareness that guides both future and current societies towards sustainable development [2].

The United Nations (UN) defines “sustainable development” on the first pages of the Brundtland report (The World Commission on Environment and Development, 1983) as “that which meets the needs of the present without compromising the ability of future generations to meet their own needs”. The action line related to this field was set out by those summits, such as the Rio de Janeiro Earth Summit (1992), which adopted Agenda 21, consisting of a number of issues to be addressed at an international, national and local level by the whole global population, with the aim of reaching sustainable development.

To be able to trace a path towards sustainability, a process of awareness of facts and anthropic actions that are carried out daily and that are damaging for the environment must first be undertaken. Subsequently, it is necessary to insist on the planning of certain actions that promote environmental enhancement [3].

If we look at young adults, a high percentage of individuals are concerned about the course our planet is taking if we do not stop these trends of pollution and consumption of natural resources. However, despite expressing this commitment, it is superficial and it is a socially or even academically imposed matter rather than a moral question [4]. Based on this, the need to implement EE as a discipline of knowledge in university degrees has been manifested over the years, especially for those degrees linked to education. EE in further studies is seen as an option to modify the relationship between nature and human beings, and therefore, it is revealed as an instrument for social transformation and empowerment, with the aim of achieving a more harmonious and equitable society [1,5,6].

Universities have tried to implement EE as an inter-disciplinary subject in their official syllabuses; for example, through the establishment of the ACES Network (Curriculum greening the Curriculum of Higher Education, acronym in Spanish), which is the result of a project involving a network in multiple Spanish and Latin American universities [7]. Despite this fact, insufficient levels of environmental literacy are seen in future teachers [8]. Indeed, it is necessary that future preschool and primary teachers are trained in EE, so that they will be able to properly share their knowledge with the future generations [9].

It is necessary for our future teachers to have the conceptual knowledge, the action lines to follow, and the ethical values and attitudes towards sustainability in order to develop actions that instill EE and its principles in young students, who will be the future citizens [3]. The matter of sustainability has already been seen in Further Education classrooms, in an effort to find out students’ attitudes towards this phenomenon, and to discover their individual specific contributions to the environment [10].

In Spain, various organizations have established sustainability indicators in Further Education institutions. One example is the Global Reporting Initiative (GRI G4 Guidelines), published back in July 2018. It includes specific guidelines for waste processing, pollution emissions and environmental compliance. These guidelines have been taken as a reference in researches for its possible application in Further Education, with the aim of making this institution a responsible social space [11,12].

Therefore, based on this theoretical paradigm, several previous pieces of research related to the analysis of human attitudes and behaviours in university students towards the environment, are recognised [13,14]. These pieces of research are about the challenges and constraints that are still barriers to be broken down, not only in Further Education, but also for society as a whole [15,16].

Firstly, it is important to highlight some studies of an evaluative nature that assessed Further Education students’ attitudes towards current environmental challenges, in particular those related to pollution and its types, and consequently, to climate change [17]. Results revealed that most students, especially the male sample, knew the phenomena of climate change and global warming, although they were not aware of the extent of the impact of some types of pollution, such as air or acoustic pollution.

In parallel, Pandve and Raut [18] found that students of Medicine stated that industrial and vehicular pollution are the types that have the greatest impact on climate change, and that apart from warnings about natural disasters that global warming brings about, the real alarm consists of the possible appearance of health problems in the population in future. This is a danger that neither young people nor society at large particularly take into account [19] and that could happen in a near future if these environmental dynamics remain.

Faced with this situation, Further Education students have questioned the approaches and measures on environmental awareness within educational disciplines, due to the scarcity of not only actions towards environmental preservation and conservation, but also national policies on EE in Further Education institutions [14].

In contrast, there are different studies analysing the impact on diverse typologies of pollution within the educational and social systems. Among them, the evaluation of acoustic pollution appears in well-known studies that analyse noise exposure in some academic centres and its relationship with the health problems present in teachers and students [20,21]. Furthermore, Secchi et al. [22] and Jahan et al. [23] support the relationship between the academic performance of students and the urban noise level around the academic centre, such as ordinary traffic, the noise from planes when flying overhead, or the noise made by trucks and heavy haulage.

Based on these challenges, Ramprasad and Subbaiyan [24] carried out an evaluation of Further Education students’ attitudes concerning the importance of acoustic pollution and its prevention, whose results showed a correlation between the absence of noise and satisfaction in class, and between the academic performance of students and their positive perceptions of the classroom as a learning space. Structural measures were proposed that involve the improvement of soundproofing in classrooms to eradicate acoustic pollution in education centres [25].

Regarding other causes of pollution, chemical pollution has been presented as related to the wide range of everyday products causing air pollution, such as aerosols, fertilisers and chemical pesticides, which consequently cause atmospheric and global warming [26]. Analyses like Anderson et al.’s [27] let us visualise the concern about non-biodegradable products, especially plastics, whose presence in various everyday objects make us participants in an increasing pollution process.

Lastly, the management of rubbish, and in particular, solid urban waste management in different urban areas, has created another key factor for the study of people’s customs, especially young people. Pieces of research go into the level of waste production of residents in different cities and its management, as well as the inhabitants’ attitudes towards this phenomenon. Likewise, it is indicated that a waste overproduction of varied nature exists, and that citizens are concerned regarding its disposal, with emphasis on the waste accumulation that is being generated in different landfill sites [28,29].

All this has been addressed from the analysis of governmental actions, with the aim of eradicating this problem. Among the solutions proposed, we can highlight the implementation of inner containers, and rubbish bags subject to charge, as well as the promotion of public information campaigns that foster waste separation and its optimal management [30]. Furthermore, the use of sustainable and recycled plastics is presented as an alternative to the use of petroleum-based plastics [31].

In view of this issue, it is indispensable to train the population about different daily actions that form an action line aimed at sustainability and the development of ecocentric attitudes towards natural spaces. Moreover, this paper intended to identify the environmental attitudes in future Preschool and Primary Education teachers towards certain habits of an environmental nature in order to set a starting point for a future action line, with the aim of leading society, especially students, towards a sustainable lifestyle.

## 2. Materials and Methods

This study is framed within a quantitative methodology, of descriptive and inferential nature, of which the aim was to find out about the attitudes of Further Education students. To achieve such an objective, this study consisted of different phases:

(1) A descriptive phase that intended to show the measures of central tendency, dispersion and shape of the data distribution. Additionally, the localisation of potential outliers and the verification of the normality of the tendency of the data depending on famous statistical tests (Lilliefors and Shapiro-Wilk tests).
(2) Based on the linearity gathered by the tests, non-parametric tests were carried out, which determined the relationship between the different variables depending on the study’s dimensions with two aims: making any observable relationships between them visible and verifying whether they show statistically significant differences.
(3) Determination of a confirmatory conceptual model by means of a SEM, in which the existent covariant relationships, either observable or non-observable, among the different variables of the study were seen. Through the realization of this model, we intended to observe the bidirectional relationships between the study variables, as well as the significant differences between the covariances, which through a unidirectional study model would not be possible. [32].

### 2.1. Participants

The population consisted of Preschool and Primary Education degree students within their second year at the University of Granada (Spain), at the Faculty of Sciences of Education in the city of Granada (Spain), for the 2018–2019 academic year. The sample was comprised of 307 students selected randomly through a process of systematic random sampling. To determine the study sample, cluster probability sampling was used [33]. Clusters were predetermined based on the group to which each student belonged. The degree of Preschool and Primary Education at the University of Granada has two and eight groups in the second year, respectively (*n* = 611). Therefore, five groups were randomly selected to get a sample size (*n* = 307), which was determined with a 95% confidence interval and a 5% margin of error.

Regarding sample features, it is important to highlight that it was made up of 25.7% men (*n* = 79), which constitutes 12.93% of the total population (*n* = 611); and 74.3% women (*n* = 228), which was 37.32% of the initial population, with an arithmetical mean of 21.05 and a standard deviation of 3.425. A majority of women is quite common among university degrees within the education sphere [34,35]. The age range was between 18 and 43 years old.

### 2.2. Instruments

The instrument used was the scale of environmental attitudes towards specific problems by Moreno, Corraliza and Ruíz [36].

The scale consists of 50 items, grouped into 10 subscales. However, we analyse 20 items (5 for each subscale) associated to the study of attitudes and environmental awareness concerning pollution, acoustic and chemical pollution and waste management. It is a four-point Likert-type scale: 1 = None, 2 = A little, 3 = Quite a lot, 4 = Completely.

This scale comprises different factors linked to both the individual and social concern of participants regarding a range of environmental issues and attitudes. The origin of these scales comes from the study of the main variables that include the main contents of the environmental crisis. Specifically, the following issues were analysed: (Table 1) [36]

- Chemical Pollution (CP): linked to awareness about the toxicity of these products (I.3), the danger they pose to the planet itself (I.1) and the awareness on how to use them. It encourages population in using natural products (I.2) or at least not so harmful ones (I.4).
- Attitudes regarding Pollution (ATT): subscale associated to specific and personal attitudes towards various behaviours in a natural environment (I.6, I.8), the causes of this problem (I.7) and the global concern that pollution pose in our planet (I.9, I.10).
- Rubbish and solid urban waste management (SUW): in relation to to the personal attitudes towards rubbish managent on a daily basis (I.11, I.13, I.15).
- Acoustic pollution (AP): this focuses on the attitudes towards daily events in which this variable (I.28, I.20) is involved, the influence of this problem in everyone’s health (I.17) and the analysis of the students perceptions towards the measures carried out by the Government against this phenomenon (I.16, I.19).

In contrast, the confidence level shown through Cronbach’s alpha coefficient was 0.860, which shows that this scale is a reliable measuring instrument. Furthermore, the validity of the questionnaire was checked through Guttman’s Split-half test, achieving a value of 0.719, which supports the previous idea, and hence, contrasts the scale’s ideal conditions for carrying out the research.

At the same time, Cronbach´s alpha reliable analysis was applicable in a classified way to deepen in the internal consistency of each subscale. The results showed optimal coefficients for the following subscales: Chemical pollution = 0.711; Attitudes regarding pollution = 0.771; Rubbish and solid urban waste management = 0.786; Acoustic pollution = 0.799.

### 2.3. Procedure

The study took place at the Faculty of Sciences of Education in the city of Granada (Spain) during the last term of 2018. Questionnaires were issued through Google Form, which allowed participants to complete them online.

The descriptive treatment of empirical data was analysed by using the statistical software “SPSS” version 23, and the creation of the structural equation model (SEM) was carried out by means of the statistical software “AMOS”, version 24. Structural equation modelling is frequently used in the context of the causal or path analysis that allows a simultaneous estimation of independent and dependent variables, which makes it possible to evaluate a multi-layer model. It is a technique that can handle a great number of endogenous and exogenous observable variables simultaneously, which are encompassed in latent variables that are linked to one another through different typologies of relationships [37].

The four latent variables were: chemical pollution (CP), acoustic pollution (AP), rubbish and solid urban waste management (SUW) and attitudes towards pollution (ATT) like [36] referenced in their scale. Each of these latent variables is comprised of five observable variables (previously specified in the items categorisation).

Subsequently, the structural model was developed, and the range of goodness-of-fit indexes was calculated. At the same time, they allowed us to find out whether the theoretical model developed fit the empirical data collected for the research.

## 3. Results

### 3.1. Statistical Descriptions

In Table 2 it is possible to observe the statistics of central tendency, dispersion and shape of the answers collected. In general, it is revealed that most of the arithmetical means, as well as the coefficients of the mode gathered, tend to the answer “quite a lot” and “completely”, indicating in some cases students’ concern about the varied environmental challenges (items 1, 2, 3, 12, 15, 17, 19, 20), together with other people’s erroneous behaviours that the subjects of the study had witnessed (items 4, 10, 11, 16). Along with them, items collecting significant means, linked to the difficulty perceived by the sample of being able to solve these issues (items 6, 19) are also noteworthy.

The variability of answers was acceptable; items 8, 14 and 18 stood out, as they tended to be homogenous. In the case of measures of shape, skewness indicates that most of the response items show negative skewness; therefore, they focused their data around the right of the mean, and thus, they showed distributions that are skewed on the left.

In the case of the coefficient of kurtosis, there was greater variability in the answers collected, which shows different cases of platykurtic curves which indicate that data are below the normal distribution (items 2, 3 and 4 among them). In parallel, it is also possible to observe many cases in which the curve presents a leptokurtic shape, and in which the answers gathered were above the normal tendency (items 1, 6 and 15).

### 3.2. Evaluation of the Linearity, Atypical Values and Correlation between Metric Variables

Based on the results of the statistic of shape and with the aim of analysing the distribution trend, Lilliefors and Shapiro-Wilk’s tests were conducted, which allowed us to evaluate the normality in the data distribution. Results of both tests revealed that the distribution trend was different from the linear trend, with a confidence interval of 99% (sig.l. < 0.01).

In line with Gutiérrez and Yepes, [38]; Perdomo [39], outliers need to be detected in the collected data, with the purpose of, through their identification, clarifying the following results for the application of the SEM model.

**Figure 1:**
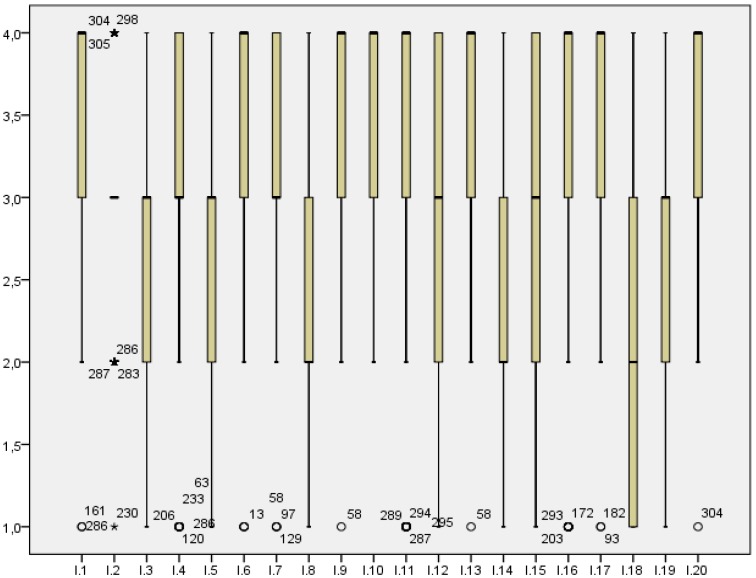
Detection of outliers in the answers collected.

Regarding atypical values in the distribution, also known as outliers, this study shows those detected in each answer item in Figure 1, in which it is possible to observe the presence of some of them in the whole range of data of each question.

The graphic in the Figure 1 shows the answers organised in quartiles, as seen in the boxes’ diagram. Each box has the major concentration of data. As for the whiskers, they represent the data with a fewer or minimal frequency. The data represented in the circles are the outliers, which were the main objective of this analysis. The number that accompanies the outliers belongs to the participant of the 307 analysed in the study.

### 3.3. Non Parametric Statistical Methods

Based on the non-normality expressed by the data distribution, the Mann-Whitney *U* test [40] was applied to examine the possible existent differences between the answers in all the descriptive categories and the gender of the group of students analysed. This test was applied with the aim of corroborating whether there are significant differences between the attitudes of men and women regarding environmental problems.

The application of Mann-Whitney *U* and Wilconxon W tests revealed a lack of significant differences regarding gender in most of the answers, except for items 6, 11, 14, 15 and 16, which reflect a differential trend regarding gender (Table 3).

### 3.4. Structural Equation Model (SEM)

Statistical fit-indexes of the model responded optimally for the study. A significant likelihood value of Chi-square was obtained (X^2^ = 299,762, df = 164; *p* < 0.0001). Nevertheless, in view of the sensitiveness of this index regarding the sample size, other standardised indexes were considered as less sensitive to this situation [41].

Next, the goodness-of-fit index (GFI) was calculated, which gave a result of 0.911, which is very close to the idyllic value (GFI = 1); in this sense, there was an excellent fit to the model developed. An amendment of the GFI is the parsimony goodness-of-fit index (PGFI), of which the coefficient obtained by the model developed was 0.682 (0.5 < x < 0.7), which is in the optimum range of fit; therefore, it supplements the vision provided by the previous goodness-of-fit index and corroborates that the model is optimal for predicting events.

The root mean square error of approximation (RMSEA) had an optimum coefficient of 0.05, which indicates an anticipated fit according to the value of the population, and hence, it fits reality.

The root mean square residual (RMR) in the model had a coefficient of 0.038, and for its proximity to zero value, it was considered as an idyllic adjustment that reveals that variances and covariances in the sample of students do not vary excessively from the estimations obtained in the model. As for the variable correlation, the expected cross-validation index (ECVI) shows a value of 0.82, which proves the correlative relationship existent between the different covariances of the study variables. In essence, the calculation of these indexes helped to conclude that the model developed fit well with the empirical data.

**Figure 2:**
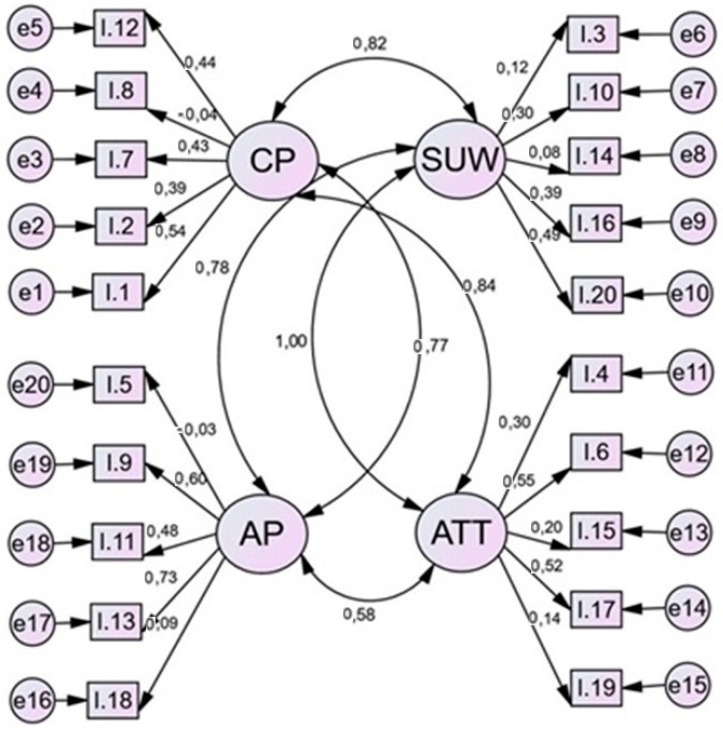
Structural equation model with regression weights. Note: CP: Chemical pollution; SUW: Solid urban waste; ATT: Attitudes towards pollution; AP: Acoustic pollution.

Obtaining the structural model allows observations to be made among the different latent variables that make up the study (Figure 2). As can be seen, the existent bidirectional relationships show optimal correlation coefficients.

Especially remarkable is the existent coefficient between the latent variable “urban solid waste and rubbish management” (USW) and the attitudes presented towards pollution (ATT) that present a coefficient close to the maximum. After these, the existent relationships between chemical pollution (CP) and the other variables are also important, showing strong correlation indexes (CP<->ATT = 0.84; CP<->USW = 0.82; CP<->AP = 0.77). Lastly, we observed that the relationship between acoustic pollution and students’ attitudes regarding pollution as a problematic issue is a weaker correlation, yet it remains significant (ATT<->AP = 0.58) (Table 4). All these estimations of values have a perfect significance, obtained through *p* value.

## 4. Discussion

Environmental challenges and, more specifically, all kind of pollution, constitute an issue for Further Education students. As the results herein reflect, students are concerned about the present situation of the planet, and describe pollution as the main environmental issue today. The results obtained by the SEM model showed that the main concern was about the rubbish and solid urban waste management, which is one of the analysed variables student encounter daily. This explains why the level of correlation is this high [14]. In line with Otsuka et al.’s study [4], the idea that there is only a true commitment to a phenomenon after ecological disasters happen is hereby corroborated. This fact is similar in different studies that state the relationship existent between the social commitment towards environmental preservation after devastating events like erosion, forest destruction or nuclear pollution [1].

Linked to this issue, we found what is considered to be the consequence of all these facts—climate change. Results of the scale, related to global and atmospheric warming of the planet, were shown among the issues that society is most concerned about. The results obtained in the chemical subscale, which was the one that focuses on the management of various products facing global warning, and its proved relation with another scale, pollution, show that the student’s concern is based on those dimensions. Undoubtedly, climate change was the environment phenomenon that generated the most concern. This is stated in studies that evaluated the same situation in Further Education [17,18].

If we move on to examining categorical variables, results reflect the importance given to as well as the existent correlation between the different environmental latent variables included in the study. The relationship between concern regarding pollution, and the management and production of urban solid waste and rubbish was the most noteworthy relationship. Furthermore, in spite of the fact that students did not define themselves as big producers of waste, they were concerned about piles of waste in city streets. Moreover, they admitted their incapability of managing and removing waste, affirmations that agree with studies such as [28] or [29]. Apart from that, attention was also drawn to the inefficient of measures undertaken by governmental authorities for waste management and disposal, a fact that matches studies that evaluated ecological policies in the same way [30].

Many of these products were chemical, a subscale that shows a strong correlation with the management of solid urban waste and rubbish. The management of chemical products and elements that emit toxic gases into the atmosphere has signified a key factor in the answers gathered. This is not only for its association to certain processes, such as global warming or environmental pollution, but because the main concern centres on health problems (certain illnesses, especially respiratory diseases) that these products and their emissions may cause the population. Undoubtedly, the lack of changes to these products, a phenomenon that this sample analysed and its free management reports, is a concern for all the individuals. Similar opinions were collected not only in this study of an academic nature, but also in clinical studies and medical education [18,26].

Polluting habits, such as travelling by car or any other vehicle, not only emits toxic gases into the atmosphere but also creates noise. Results of this study showed an average correlation between the attitudes and the awareness of students towards pollution and acoustic pollution. According to similar studies, the continuous exposure to noise affects our health; therefore, this issue must be addressed with the same emphasis as the other typologies of pollution, considering that it has a direct impact on the daily life of every human being [20].

Thus, factors experienced by students in their daily lives, such as traffic and noise, at home or in schools, were presented as a need to be improved according to other pieces of research [22]. Transport noise, such as motorcycles, constitute an inconvenience that has already been evaluated [23], and which is corroborated in our study. Given this situation, the main answers collected regarding this aspect point at the requirement for governmental administrations to propose different organisational measures that promote a significant improvement in the noise decrease in urban areas, which leads to the population’s wellbeing [25].

Consequently, and based upon these results, Environmental Education throughout students’ university careers is essential [2,6]. Training in this field is necessary to foster ecocentric attitudes towards the environment among students, which beat a path towards environmental sustainability, due to the fact that current generations in Further Education are the future of this society.

Finally, the study agrees with the statements used by Blanco et al. [15], in which the main ecological associations and organisations must view their action lines in Further Education as an institution that can enhance and promote a change in attitude and set up a sustainable trend in present and future society. In this regard, it will be necessary to establish different nodal networks among the different universities and higher institutions by means of collaborative virtual settings, with the aim of setting a linear and rigorous action line, underpinned by responsible policies that support the cause [5,42].

## 5. Conclusions

Environmental Education is presented as a priority to continue strengthening within Further Education, understood as entrepreneurial training that will promote a change at a social level among the population. In parallel, Further Education is presented not only as a major educational organism, but also as a decision-making institution that collaborates with governmental authorities when it is time to make decisions in educational spheres related to sustainability and care for the environment. The results in this study back up the importance of making students aware of the seriousness of pollution, its repercussions for the health of the planet and for students’ own health.

Therefore, from this study, we intend to foster carrying out similar evaluations regarding environmental components or issues to different groups of students from different academic phases and disciplines, with the aim of continuing to promote a process of environmental awareness among society and, more specifically, among young people in education. Based on studies of this nature, it will be possible to turn into a research path aimed at undertaking specific actions that try to reduce the different typologies of pollution described above.

As seen in this study, the different problematic variables described are constantly related to one another, and hence, any actions addressed to reducing them will lead to a collective improvement that aims to create ecocentric people, who have a feeling of responsibility and care towards the present natural environment, in order to value it and preserve it for the future [7,43,44].

There is still a long way to go within the implementation of Environmental Education, in which Further Education plays a key role in the awareness, education and ecological actuation of future teachers, who are the ones who are going to share their concerns with the future generations. However, every step forward is a victory.

## Figures and Tables

**Table 1:** Relationship between items and the descriptive category of the scale it belongs to [36].

Items	Subscale
1. The planet is so polluted by chemical products that it has become a threat for health.	Chemical pollution
2. People surrounding me use plenty of products that pollute the environment.	
3. Every year new chemical products are released without prior analysis of their effects.	
4. I do not buy organic food (without fertilisers/pesticides), since they are more expensive, or more difficult to find.	
5. I feel guilty for using non-biodegradable cleaning products, given that I contribute to polluting the environment.	
6. People around me only protest regarding the environment when ecological disasters happen.	Attitudes regarding pollution
7. The increase in the atmospheric temperature is caused by the increasing and continuous use of fossil fuels (coal, oil …).	
8. I would be inflexible concerning punishing environmental pollution offences.	
9. To me pollution seems to be the most serious environmental issue.	
10. It is really hard to reduce pollution to protect the environment.	
11. There is no capacity nowadays for an integrated management of solid urban waste.	Rubbish and solid urban waste management
12. People throw rubbish on the ground when nobody is watching.	
13. I do not know how to generate less rubbish.	
14. When I see someone dropping rubbish, I feel like reprimanding that person.	
15. The waste accumulation from cities is a really serious issue.	
16. Administrations lack adequate means to reduce city noise.	Acoustic pollution
17. Acoustic pollution in cities is damaging for human health.	
18. When I hear very noisy motorists, I feel like reprimanding them.	
19. Local governments should put greater emphasis on noise reduction and restriction.	
20. Locals in my area frequently complain about noise.	

**Table 2:** Statistical descriptions of responses collected.

Items	Mean	Mode	SD	Skewness	Kurtosis
I.CP.1	3.57	4	0.586	−1.219	1.509
I.CP.2	3.01	3	0.685	−0.78	−0.676
I.CP.3	3.11	3	0.711	−0.333	−0.439
I.CP.4.	2.47	3	0.997	0.015	−1.05
I.CP.5.	2.96	3	0.801	−0.433	−0.271
I.A.1.	3.13	3	0.796	−0.641	−0.119
I.A.2.	3.48	4	0.628	−1.060	1.177
I.A.3.	3.31	4	0.849	−1.081	0.346
I.A.4.	3.47	4	0.606	−0.891	0.705
I.A.5.	2.77	3	0.867	−0.177	−0.709
I.USW.1	3.13	3	0.796	−0.066	−0.488
I.USW.2	3.53	4	0.622	−0.991	−0.062
I.USW.3	2.49	2	0.961	0.025	−0.944
I.USW.4	3.31	4	0.849	−1.081	0.346
I.USW.5	3.67	4	0.533	−1.527	2.175
I.AP.1	2.52	3	0.977	−0.039	−0.911
I.AP.2	3.54	4	0.566	−0.894	0.416
I.AP.3	3.19	4	0.971	−0.924	−0.319
I.AP.4	3.44	4	0.651	−0.835	−0.053
I.AP.5	1.89	1	0.950	0.646	−0.745

Note: SD = Standard deviation. CP: Chemical pollution; SUW: Solid urban waste; AP: attitudes towards pollution; AP: Acoustic pollution.

**Table 3:** Conduction of non-parametric tests item-sex and its level of significance.

Item-Gender		Average Range	Sum of Ranges	U Mann-Whitney	W. Wilcoxon	Z	P
I.1	Male	150.11	11,859	8699	11,859	−0.531	0.596
	Female	155.35	35,419				
I.2	Male	150.67	11,903	8743	11,903	−0.428	0.669
	Female	155.15	35,375				
I.3	Male	159.99	12,639	8533	8533	−0.746	0.456
	Female	151.93	34,639				
I.4	Male	154.31	12,190.50	8981	8981	−0.039	0.969
	Female	153.89	35,087.50				
I.5	Male	150.90	11,921	8761	8761	−0.376	0.707
	Female	155.07	35,357				
I.6	Male	146.39	11,564.50	7804	7804	−2.007	0.044
	Female	156.64	35,713.50				
I.7	Male	142.85	11,285.50	8125	8125	−1.419	0.156
	Female	157.86	35,992.50				
I.8	Male	164.47	12,993	8179	8179	−1.265	0.206
	Female	150.37	34,285				
I.9	Male	154.58	12,212	8960	8960	−0.078	0.938
	Female	153.80	35,066				
I.10	Male	150.37	11,879	8719	8719	−0.489	0.625
	Female	155.26	35,399				
I.11	Male	137.35	10,851	7691	7691	−2.103	0.035
	Female	159.77	36,427				
I.12	Male	146.36	11,562.50	8402	8402.50	−0.958	0.338
	Female	156.65	35,715.50				
I.13	Male	145.40	11,486.50	8326	8326.50	−1.123	0.261
	Female	156.98	35,791.50				
I.14	Male	172.63	13,638	7534	7534	−2.261	0.024
	Female	147.54	33,640				
I.15	Male	172.85	13,655	7517	7517	−2.325	0.020
	Female	147.47	33,623				
I.16	Male	129.81	10,255	7095	7095	−3.099	0.002
	Female	162.38	37,023				
I.17	Male	141.65	11,190	8030	8030	−1.631	0.103
	Female	158.28	36,088				
I.18	Male	167.56	13,237	7934	7934.50	−1.680	0.093
	Female	149.30	34,040				
I.19	Male	151.94	12,003	8843	8843	−0.254	0.800
	Female	154.71	35,275				
I.20	Male	144.32	11,401.50	8241	8241.50	−1.419	0.156
	Female	157.35	35,876.50				

Note: Z = Wilcoxon statistic; P: *p* value.

**Table 4:** Weights and standardised regression weights.

Items	R.W			SRW	
	Estimations	SE	CR	Estimations	*p* Value
ICP1	1.00			0.539	
ICP2	0.857	0.170	5.053	0.395	***
ICP3	0.960	0.180	5.347	0.426	***
ICP4	−0.112	0.214	−0.522	−0.035	0.042
ICP5	1.119	0.204	5.475	0.441	***
ISUW1	1.00			0.117	
ISUW2	1.936	1.153	1.679	0.296	0.039
ISUW3	0.806	0.800	1.008	0.080	0.032
ISUW4	3.477	2.009	1.731	0.389	0.047
ISUW5	2.764	1.572	1.758	0.492	0.019
IA1	1.000			0.300	
IA2	1.446	0.359	4.026	0.551	***
IA3	0.711	0.290	2.447	0.197	0.022
IA4	1.316	0.332	3.970	0.519	***
IA5	0.507	0.271	1.872	0.140	0.041
IAP1	−0.324	0.848	−0.382	−0.027	0.044
IAP2	4.198	3.319	1.265	0.600	***
IAP3	5.719	4.546	1.258	0.477	***
IAP4	5.875	4.636	1.267	0.730	0.022
IAP5	1.000			0.085	

Note: RW = Regression Weights, SRW = Standardised Regression Weights; SE = Estimation of Error; CR = Critical Ratio. ICP: Item chemical pollution; ISUW: Item solid urban waste; IA: Item attitudes towards pollution; IAP: Item acoustic pollution.
